# Global tuberculosis targets and milestones set for 2016–2035: definition and rationale

**DOI:** 10.5588/ijtld.17.0835

**Published:** 2018-07-01

**Authors:** K. Floyd, P. Glaziou, R. M. G. J. Houben, T. Sumner, R. G. White, M. Raviglione

**Affiliations:** * Global Tuberculosis Programme, World Health Organization, Geneva, Switzerland; † Department of Infectious Disease Epidemiology, London School of Hygiene & Tropical Medicine, London, UK

**Keywords:** TB, control, strategy

## Abstract

**>BACKGROUND:**

Global tuberculosis (TB) targets were set as part of the World Health Organization's End TB Strategy (2016–2035) and the Sustainable Development Goals (2016–2030).

**>OBJECTIVE:**

To define and explain the rationale for these targets.

**>DESIGN:**

Scenarios for plausible reductions in TB deaths and cases were developed using empirical evidence from best-performing countries and modelling of the scale-up of under-used interventions and hypothetical TB vaccines. Results were discussed at consultations in 2012 and 2013. A final proposal was presented to the World Health Assembly in 2014 and unanimously endorsed by all Member States.

**>RESULTS:**

The 2030 targets are a 90% reduction in TB deaths and 80% reduction in TB incidence compared with 2015 levels. The 2035 targets are for reductions of 95% and 90%, respectively. A third target—that no TB-affected households experience catastrophic costs due to the disease by 2020—was also agreed.

**>CONCLUSION:**

The global TB targets and milestones set for the period 2016–2035 are ambitious. Achieving them requires concerted action on several fronts, but two things are fundamental: 1) progress towards universal health coverage to ensure that everyone with TB can access high-quality treatment; and 2) substantial investment in research and development for new tools to prevent TB disease among the approximately 1.7 billion people infected.

## Abstract (FR)

**>CONTEXTE:**

Les objectifs mondiaux relatifs à la tuberculose (TB) ont été fixés dans le cadre de la stratégie Halte à la TB (2016–2035) de l'Organisation Mondiale de la Santé et des Objectifs de Développement Durable (2016–2030).

**>OBJECTIF:**

Définir et expliquer la justification de ces objectifs.

**>SCHÉMA:**

Les scénarios de réduction plausible des décès par TB et de cas de TB ont été élaborés grâce aux preuves empiriques des pays les plus performants et par modélisation de l'accélération des interventions sous-utilisées et d'hypothétiques vaccins contre la TB. Les résultats ont été discutés lors de consultations en 2012 et 2013. Une proposition finale a été présentée à l'Assemblée Mondiale de la Santé en 2014 et adoptée à l'unanimité par tous les États Membres.

**>RÉSULTATS:**

Les objectifs 2030 sont une réduction de 90% des décès par TB et une réduction de 80% de l'incidence de la TB, comparés aux niveaux de 2015. Les objectifs 2035 sont une réduction de 95% et 90%, respectivement. Un troisième objectif—qu'aucun foyer affecté par la TB ne subisse des coûts catastrophiques dus à la maladie d'ici 2020—a également été approuvé.

**>CONCLUSION:**

Les objectifs et étapes mondiaux relatifs à la TB établis pour la période 2016–2035 sont ambitieux. Les atteindre requiert une action concertée sur de nombreux fronts, mais deux choses sont fondamentales : le progrès vers la couverture santé universelle afin d'assurer que toute personne atteinte de TB puisse accéder à un traitement de grande qualité, et un investissement substantiel dans la recherche et le développement de nouveaux outils de prévention de la TB maladie parmi les 1,7 milliards environ de personnes infectées.

## Abstract (SP)

**>MARCO DE REFERENCIA:**

Las metas mundiales de la tuberculosis (TB) se adoptaron como parte de la Estrategia Fin a la Tuberculosis (2016–2035) de la Organización Mundial de la Salud y los Objetivos de Desarrollo Sostenible (2016–2030).

**>OBJETIVO:**

Definir y explicar el fundamento lógico de estas metas.

**>MÉTODO:**

Las hipótesis de posibles disminuciones de las defunciones por TB y de los casos de TB se formularon a partir de datos empíricos de los países con mejor desempeño, modelizaciones de la ampliación de escala de las intervenciones subutilizadas y de la repercusión de eventuales vacunas antituberculosas. Los resultados se analizaron en consultas realizadas en el 2012 y el 2013. En el 2014 se presentó una propuesta final a la Asamblea Mundial de la Salud, que recibió la aprobación unánime de los Estados Miembros.

**>RESULTADOS:**

Las metas para el 2030 consisten en la disminución de un 90% de las defunciones por TB y la disminución de un 80% de la incidencia de la enfermedad, con respecto a la situación del 2015. Las metas de reducción en el 2035 son 95% y 90%, respectivamente. Se acordó además una tercera meta que consiste en que ninguna familia afectada por la TB asuma costos catastróficos debido a la enfermedad hacia el 2020.

**>CONCLUSIÓN:**

Las metas y los hitos mundiales de la TB establecidos para el periodo del 2016 hasta 2035 son ambiciosos. Alcanzarlos exige una acción concertada en muchos frentes, pero existen dos elementos fundamentales, a saber: el progreso hacia la cobertura sanitaria universal, de manera que todas las personas con TB tengan acceso a un tratamiento de gran calidad, e inversiones apreciables en investigación y en el desarrollo de nuevos instrumentos para prevenir la evolución hacia la enfermedad tuberculosa en todas las personas infectadas que son cerca de 1700 millones.

AT THE TURN of the twenty-first century, the United Nations (UN) established eight Millennium Development Goals (MDGs) and associated targets for 2015. These were endorsed by all countries and became the focus of national and international development efforts (www.un.org/millenniumgoals). Within this framework, three targets for reductions in the tuberculosis (TB) disease burden were set: incidence should be falling by 2015, and prevalence and mortality rates should be halved by 2015 compared with 1990 levels. The World Health Organization's (WHO's) Stop TB Strategy 2006–2015 was designed to achieve these targets.^1^ The WHO published its assessment of whether the targets were achieved in its 2015 global TB report; incidence was estimated to have been falling at an average of 1.5% per year since 2000, and prevalence and mortality rates were assessed to have fallen by respectively 47% and 42% compared with the levels in 1990.^2^

Work on post-2015 UN goals and targets began in 2012, and 17 Sustainable Development Goals (SDGs) for 2030 were agreed in September 2015.^3^ One of the SDGs is to ‘Ensure healthy lives and promote well-being for all at all ages’, under which a target is to ‘End the epidemics of AIDS, TB, malaria and neglected tropical diseases, and combat hepatitis, water-borne diseases and other communicable diseases’. In this context, the WHO initiated the development of a post-2015 global TB strategy and targets in 2012.

Following 2 years of consultations, the new strategy, now known as the End TB Strategy and covering the period 2016–2035, was endorsed by the World Health Assembly in 2014.^4^,^5^ The overall goal is to ‘End the global TB epidemic’, and ambitious targets for reductions in TB deaths and cases have been set for 2030 (the SDG end date) and 2035. The present article defines and explains the rationale for these targets and associated milestones set for 2020 and 2025.

## METHODS

Target setting was underpinned by seven principles (Table 1) consistent with those used for the SDGs.^6^ The number of TB deaths and the TB incidence rate (new cases per 100 000 population per year) were selected as the two most important indicators for which targets should be set.

**Table 1:**
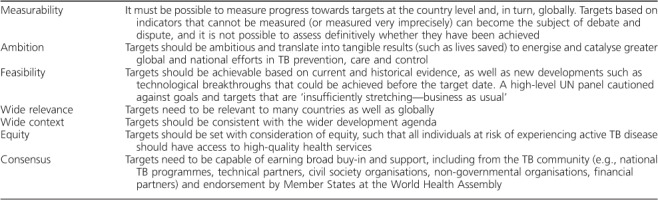
Principles underpinning post-2015 global TB targets

**Figure 1:**
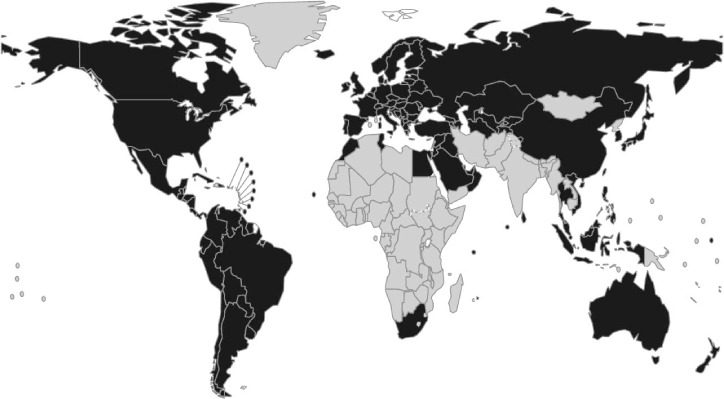
Availability of vital registration data. Countries shown in black are those in which TB mortality could be measured directly from vital registration data in 2015. TB = tuberculosis.

The number of TB deaths is directly measurable at country level via national vital registration systems, in which causes of death are recorded using standard international coding systems; 128 countries had such systems in 2015 (Figure 1), and they could be introduced elsewhere. Targets for reductions in TB deaths can be more ambitious than those for TB cases, as mortality can fall faster than disease incidence if both incidence and the proportion of TB cases who die from the disease (case fatality ratio [CFR]) decline. Reductions in TB deaths can also be linked to equity: whatever the number of cases, all countries can aim to reach the same low CFR based on universal health coverage (UHC), i.e., access for all to essential preventive and treatment health care interventions, with financial protection.^7^,^8^

TB incidence was selected as an indicator for measuring reductions in the number of cases of TB disease. Although incidence was estimated with considerable uncertainty in most countries in the MDG era,^2^ notifications of TB cases to national authorities provide a good proxy if there is limited under-reporting of detected cases, limited under-diagnosis and limited misdiagnosis. Underreporting, underdiagnosis and misdiagnosis can be addressed by strengthening national surveillance and health systems. The alternative indicator, TB prevalence, was considered unsuitable because it will not be measured directly in most countries after 2015. As the burden of TB disease falls, the sample size required for national prevalence surveys become prohibitively expensive and logistically challenging.^9^

To define plausible scenarios for the reductions in TB deaths and incidence that could be achieved between 2015 and 2035, two periods were considered: 2015–2025 and 2026–2035. The status of the pipelines for new TB diagnostics, drugs and vaccines suggested that no major breakthroughs will occur during this first period.^2^ A new TB vaccine or equivalent treatment for latent tuberculous infection could become available in the second period.

**Figure 2:**
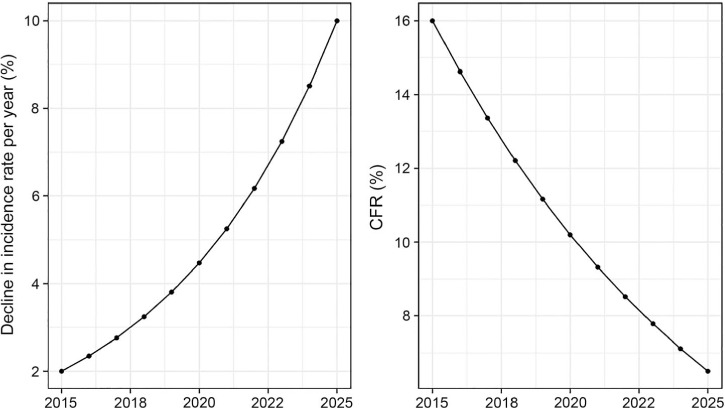
Assumed change in TB incidence rate (annual rate of decline) and the CFR, 2015–2025. The trajectory assumes that the effect of progress towards universal health coverage (and implementation of new diagnostics and drugs currently in the pipeline) intensifies around 2020 and achieves full potential by 2025. To achieve further improvements, new tools such as a post-exposure vaccine would be required. CFR = case-fatality ratio; TB = tuberculosis.

Reductions in TB deaths are driven by two factors: the annual rate at which TB incidence falls, and changes in the CFR. Illustrative scenarios for the reductions in TB deaths that could be achieved by 2025 were constructed for different combinations of these variables, allowing for projected growth in population.^10^ The assumed trajectories for changes in the incidence rate and the CFR are shown in Figure 2.

**Figure 3:**
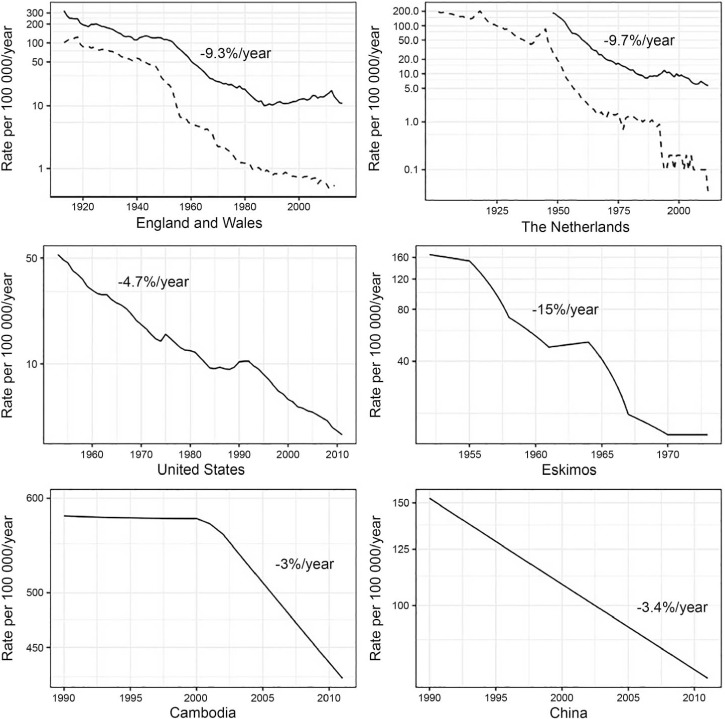
Long-term TB incidence trends in countries with robust surveillance data. At the national level, the best historical declines in TB incidence (solid line) reached about 10%/year (England and Wales, The Netherlands), while faster declines were observed in subpopulations (in Alaska among the Eskimo population). Current best-performing countries show a more modest decline of 3–5%/year (Cambodia, China). The current global decline rate is 2%/year. The dashed line in the top panels denotes TB mortality rates. TB = tuberculosis.

A ‘plausibility zone’ for targets was defined based on historic evidence about the speed at which the TB incidence rate can fall and the lowest levels of the CFR observed in settings in which the coverage and quality of anti-tuberculosis treatment was high. The limit for the decline in incidence was set at 10% per year. This is the best-ever performance historically at the national level, achieved between the 1950s and 1970s in parts of Western Europe (Figure 3) in the context of rapid socio-economic development, UHC and the introduction of chemotherapy.^11^ Faster declines have only been documented in the 1950s and 1960s, in subpopulations with incidence rates ten times the 2015 global average.^12^,^13^ The global CFR limit was defined as 6.5%, the 2013–2014 average in high-income countries.

The plausibility zone reflected historic performance without allowing for the possibility of a hypothetical scale-up of two interventions: mass screening for tuberculous infection and TB disease, followed by treatment for disease and isoniazid preventive therapy (IPT) (hereafter mass TB screening [MTS]). In the MDG era, such mass campaigns were very limited, but were considered to explore the potential reductions in TB burden that could be achieved. The potential impact of MTS was explored using a simple dynamic transmission model similar in structure to other published models.^14^,^15^ The negative consequences of MTS (number of false-positive individuals treated for infection or disease and deaths associated with the side effects of IPT) were also quantified (see Appendix).^*^

For 2026–2035, further modelling was undertaken to explore the impact of a technological breakthrough. For practical purposes, the analysis focused on the potential impact of a new vaccine with 60% efficacy, introduced in 2025, providing protection for at least 10 years and achievement of 90% effective coverage by 2035 (see Appendix).

Global consultations were held to inform the development of the End TB Strategy in 2012 and 2013, two of which were especially important. The first, in February 2013, considered the analyses described above to reach consensus on targets/milestones for 2025.^16^ The second, in June 2013, considered the recommendations of the February 2013 consultation, results of the modelling work up to 2035 and associated target proposals for 2030 and 2035 that would correspond to the goal of ending the global TB epidemic.^17^

No ethical approval was required for this work.

## RESULTS

**Figure 4:**
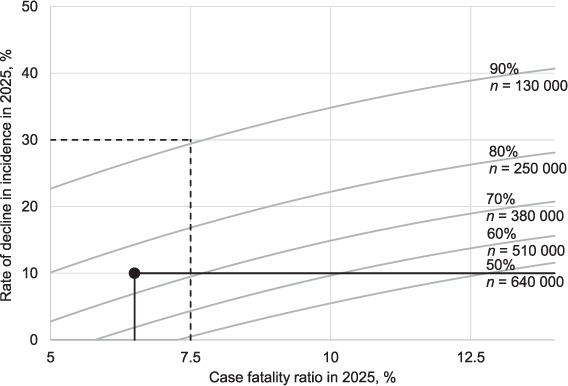
Scenarios for reductions in TB deaths that could be achieved by 2025, and the associated ‘target zone’. Contours show combinations of annual percentage declines in incidence in 2025 (y-axis) and CFR in 2025 required to produce the corresponding reductions in TB deaths. For example (dashed lines), a 90% reduction in the number of TB deaths (to 130 000 by 2025) could be achieved if TB incidence declines at 30% per year and the CFR is reduced to 7.5% by 2025. The solid black lines illustrate the plausible zone based on previously observed declines and the average CFR in high-income countries. The circle marks the most ambitious scenario within the plausible zone. CFR = case-fatality ratio; TB = tuberculosis.

The combinations of reductions in TB incidence and the CFR that would be required for reductions in TB deaths ranging from 50% to 90% by 2025 (compared with 2015) are shown in Figure 4. The plausibility zone for targets that could be reached by 2025 is shown by solid black lines. If recent trends continued (bottom right corner), the number of TB deaths would fall by about 18% between 2015 and 2025. In the most optimistic scenario, in which incidence decreases at 10% per year by 2025 and the CFR falls to 6.5% (circle), a 75% reduction in the number of TB deaths would be achieved.

**Figure 5:**
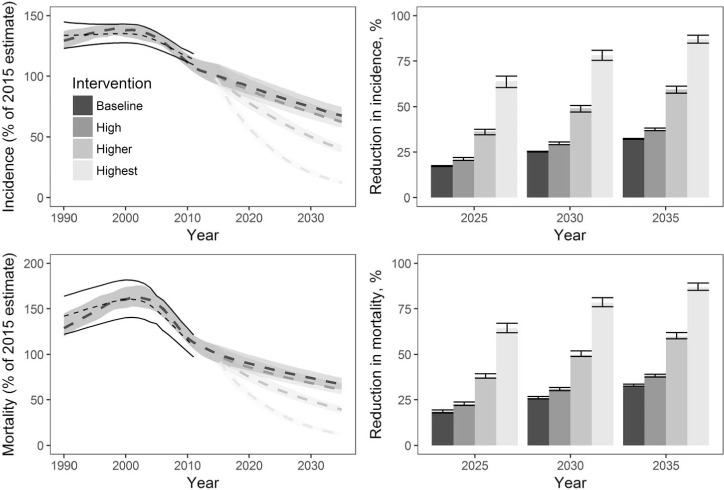
Trends and reductions in TB incidence and mortality as a result of a theoretical expansion of screening and treatment for active TB disease and latent tuberculous infection (mass TB screening [MTS])—2% background decline scenario. Left panels show the trends in TB incidence (top) and mortality (bottom), assuming the background decline in TB incidence remains at 2% per year and the CFR at 16%. Black lines show WHO-estimated incidence, dashed lines show the median model output, and shaded areas the 95% credible intervals (all values are relative to the 2015 estimated incidence). Right panels show the percentage reduction in incidence (top) and mortality (bottom) compared with 2015. Four scenarios are considered: no MTS (baseline); 5% of population screened per year, 10% completion of IPT (high); 10% screened, 50% completion (higher); 20% screened, 90% completion (highest). TB =tuberculosis; CFR = case-fatality ratio; WHO = World Health Organization; IPT = isoniazid preventive therapy.

The dynamic model suggested that with a background of a 2% annual decline in TB incidence and a CFR of 16% combined with the MTS intervention, the number of TB deaths could fall by 22–65% by 2025 (Figure 5). With the most optimistic background scenario (CFR 6.5%, annual incidence decline 10%/year by 2025), the incremental impact of the MTS intervention would be lower and the total number of TB deaths could fall by 77–90% by 2025 (see Appendix). MTS may also result in considerable undesirable effects and over-treatment, with 81–93% of those provided with anti-tuberculosis treatment not having TB.

**Figure 6:**
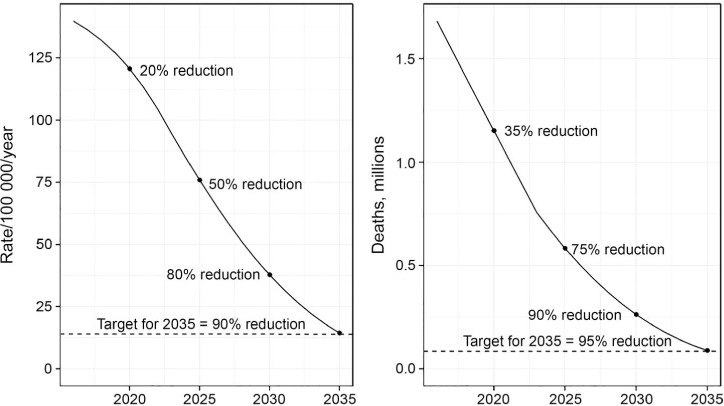
Projected TB incidence and mortality curves to reach targets and milestones, 2015–2035, assuming the annual decline in incidence reaches 10%/year, CFR is reduced to 6.5% by 2025 and the availability of an efficacious vaccine after 2025. TB=tuberculosis; CFR=case-fatality ratio.

The median trajectories for declines in TB deaths and incidence that could be achieved by 2035, assuming a technological breakthrough (i.e., vaccine) by 2025 building on a 75% reduction in TB deaths between 2015 and 2025 are shown in Figure 6. An incidence rate of around 14/100 000 (comparable with levels found in countries considered to have a low TB burden in recent years) and a reduction in TB deaths of around 95% could be achieved by 2035.

In February 2013, agreement was reached on two targets for 2025: a 75% reduction in TB deaths and a 50% reduction in TB incidence, compared with 2015 levels. Such reductions, and in particular the underlying requirement that the CFR should fall to 6.5% by 2025, implicitly require that all people with TB can access diagnosis and treatment, i.e., UHC is in place. A third high-level target linked to UHC was therefore proposed: by 2020, no TB-affected households should suffer catastrophic costs as a result of TB. In June 2013, following extension of the modelling work described above, the targets proposed for 2025 were rephrased as milestones, and 2030 and 2035 targets corresponding to the end dates of the SDGs and End TB Strategy were proposed (Table 2). The targets and milestones shown in Table 2 were endorsed by all 194 Member States at the 2014 World Health Assembly.^4^,^5^

**Table 2:**
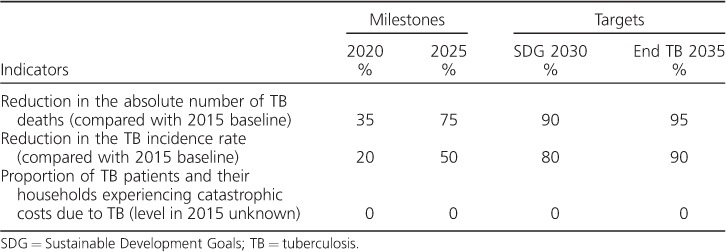
The End TB Strategy's three high-level global indicators and associated targets (2030 and 2035) and milestones (2020 and 2025)

## DISCUSSION

The global TB targets and associated milestones set by the WHO's End TB Strategy call for a 90% reduction in TB deaths by 2030 (compared with 2015) and a 95% reduction by 2035, with corresponding reductions of respectively 80% and 90% in the TB incidence rate. By 2025, TB deaths should be reduced by 75%, and by 2020 no TB patients and their households should face catastrophic costs due to TB.

The targets are ambitious, but within the limits of plausibility, and are consistent with the 2030 SDG targets for ending the epidemics of major infectious diseases, including TB, and achieving UHC. Comparable targets have also been set in the post-2015 strategies for HIV and malaria: a 90% reduction in the malaria death rate by 2030 compared with 2015 and a 90% reduction in deaths due to the acquired immune-deficiency syndrome by 2030 compared with 2010. The TB targets are measurable and promote equity, necessitating that all people who develop TB have the same high chance of receiving appropriate care and the same low chance of dying from the disease. They are also based on consensus, having earned unanimous endorsement by UN Member States at the 2014 World Health Assembly and wide buy-in from funding agencies, technical partners and civil society.

The technical work that informed the target setting was grounded in empirical evidence about the two key variables that can drive reductions in TB burden: the annual rate at which it is possible to reduce TB incidence and the proportion of cases that die from TB if there is universal access to high-quality diagnosis and treatment. They were also based on up-to-date information about the development pipelines for new TB diagnostics, drugs and vaccines, with post-2025 projections allowing for technological breakthroughs that could occur within one decade and greater use of currently under-used interventions.

To reach these targets, progress is required on several fronts,^5^ but two things are fundamental. First, UHC for essential health care services, including detection and treatment of TB, must be achieved by 2025. The 2025 milestone of reducing TB deaths by 75% requires cutting the CFR to 6.5% (the level of high-income countries), which implicitly means that all those with TB disease (both drug-susceptible and drug-resistant, and both adults and children) can access high-quality treatment. There is growing momentum to promote UHC and monitor progress towards it.^7^,^8^,^18^,^19^ The 10% per year fall in incidence that is needed by 2025 has previously been achieved only in the wider context of UHC and broader socioeconomic development, including social protection: Western Europe in the 1950s and 1960s is the best example. Similar improvements in socio-economic status, poverty reduction and improvements in living conditions in low-income countries that have the greatest burden of TB will play a key part in reaching the TB targets. Social protection mechanisms are also essential to ensure that TB patients and their households do not incur catastrophic costs, for example due to lost income from time away from work. The second fundamental requirement is a technological breakthrough by 2025 that will allow an unprecedented acceleration in the rate at which TB incidence falls between 2025 and 2035. This will happen only with substantial investment in research and development, so that new tools to substantially lower the risk of developing TB among people who are already infected can be developed.

Achievement of the targets for reductions in TB deaths and incidence at the global level does not mean that all countries need to make progress at the same pace. The strategy recognises that countries will need to make adaptations to the overall targets. The WHO has issued guidance that includes 10 priority operational indicators and associated targets that should be reached by 2025 at the latest, and recommendations for how to set country-specific targets for 2020 and 2025.^20^ In addition, the Global Plan to End TB produced by the Stop TB Partnership provides a roadmap for countries working towards the 2020 milestones. Progress in the countries with the highest burden, such as China, India, Indonesia, Nigeria, Pakistan, the Philippines and South Africa, which collectively accounted for two thirds of estimated incident cases in 2015, will strongly influence whether global targets can be achieved.

All of the indicators for which post-2015 global TB targets have been set are measurable. However, direct measurement of TB deaths and TB incidence (as opposed to indirect estimation reliant on modelling and expert opinion) will require the strengthening of routine information systems in many countries. Guidance exists on how to assess the capacity of national notification and vital registration systems to provide direct measurements of TB cases and deaths, respectively, and to use results to close identified gaps.^21^ Guidance on the measurement of catastrophic costs using special surveys has been developed.^22^ The WHO and the World Bank plan to issue a biennial report on progress towards UHC from 2015 onwards.^19^ Strengthening health information systems, in particular civil and vital registration systems, is already a prominent part of the post-2015 health agenda.^23^

The 2035 targets set within the End TB Strategy define the end of the global TB epidemic. Following endorsement by all UN Member States at the World Health Assembly, intensified action at national and global levels to operationalise the strategy is imperative.

## Figures and Tables

**Figure A.1:**
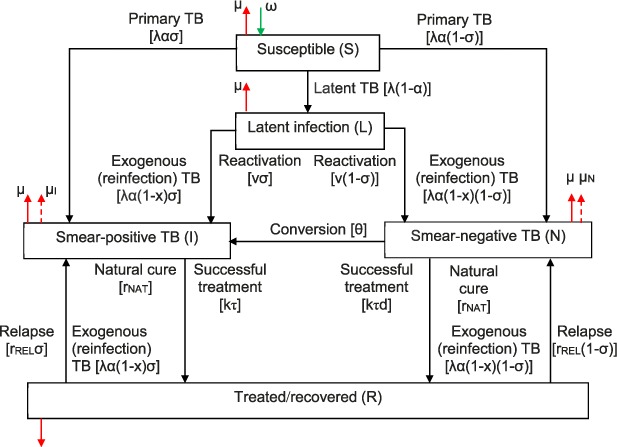
Schematic of the transmission model. Black arrows = transitions between TB states; green arrows = births; red solid arrows = background deaths; red dashes = TB deaths. TB = tuberculosis.

**Table A.1:**
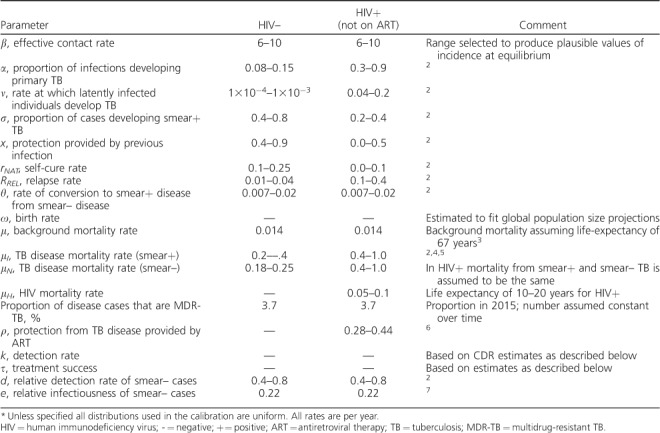
Model parameters ^*^

**Figure A.2:**
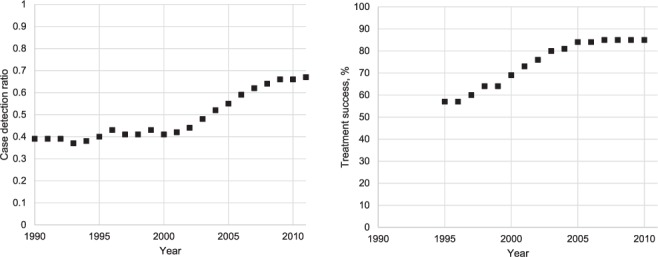
Case detection ratio and treatment success values used in the model. Values were assumed to remain at their current levels from 2011 onwards.

**Figure A.3:**
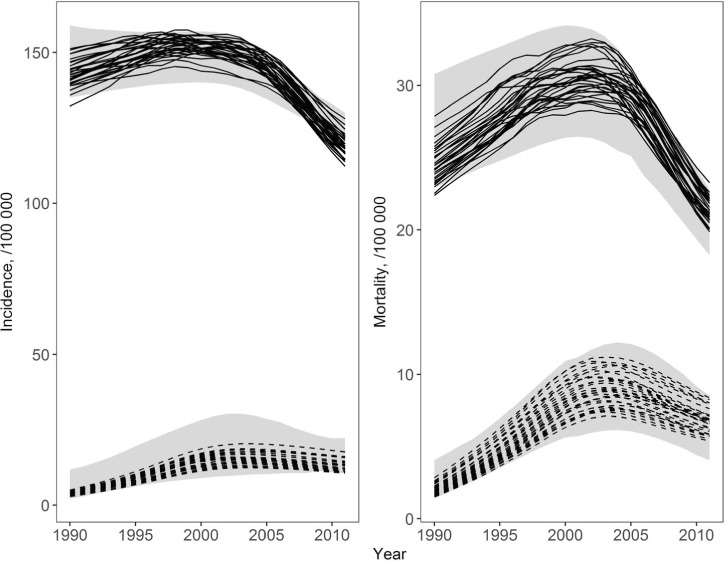
A sample of 100 model outputs generated using the calibration procedure. Upper lines=all TB; lower lines=HIV-positive TB. The shaded grey areas show the uncertainty boundaries on World Health Organization estimates.

**Figure A.4:**
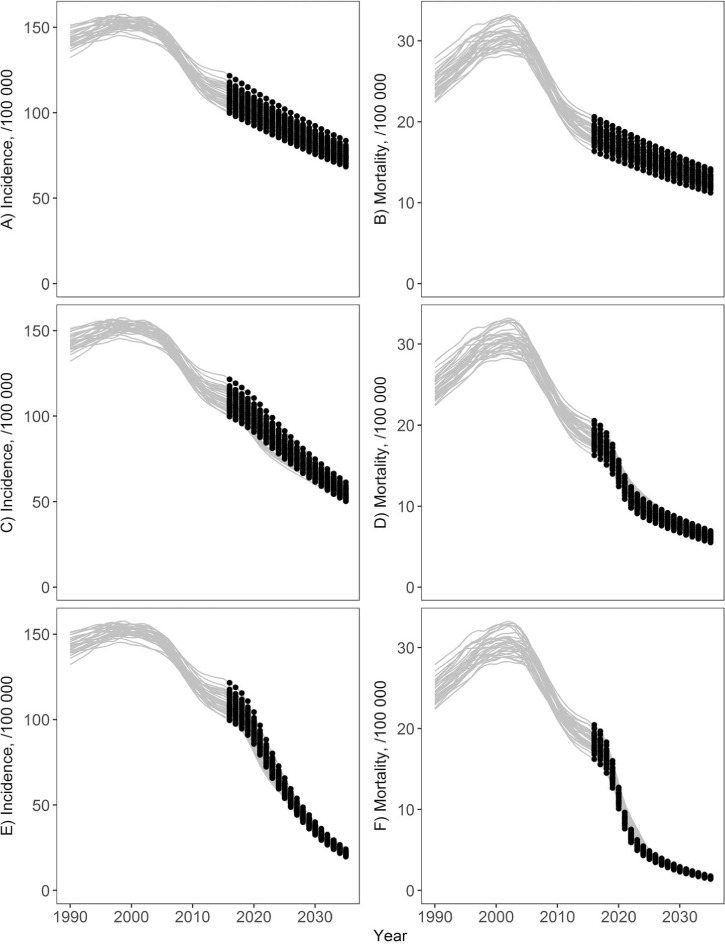
Fit of the model to the baseline declines in incidence (left) and mortality (right) from 2015 to 2025. A), B) Annual decline in incidence remains at 2%, CFR remains at 16%; C), D) annual decline in incidence increases to 4% and CFR declines to 10% by 2025; E), F) annual decline in incidence increases to 10% and CFR declines to 6.5% by 2025. Black circles = target incidence and mortality predicted by the statistical model, black lines =output of the transmission model. CFR = case-fatality ratio.

**Figure A.5:**
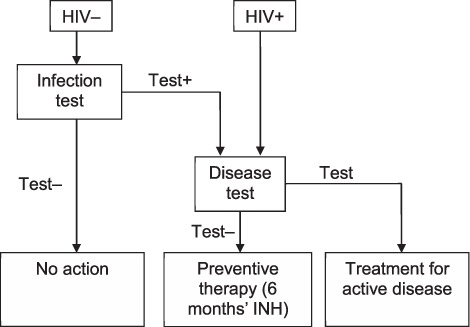
Mass screening and treatment intervention algorithm. HIV = human immunodeficiency virus; −; = negative; + = positive; INH = isoniazid.

**Table A.2:**
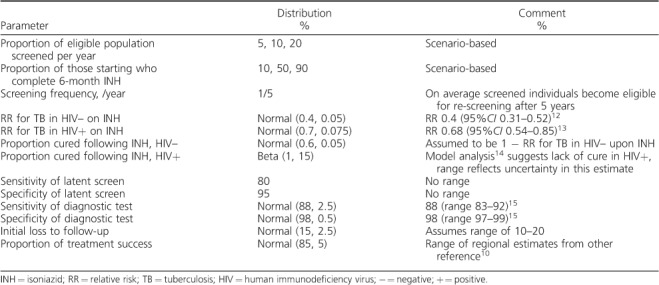
Intervention parameters

**Figure A.6:**
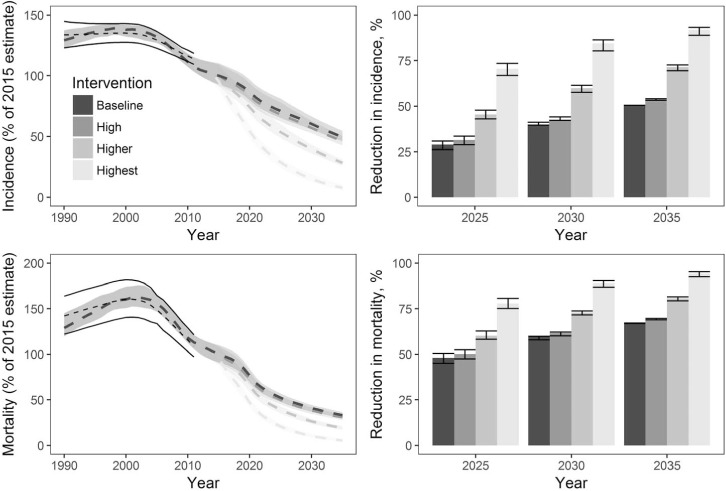
Impact of mass screening for tuberculosis in the 4% decline scenario.

**Table A.3:**
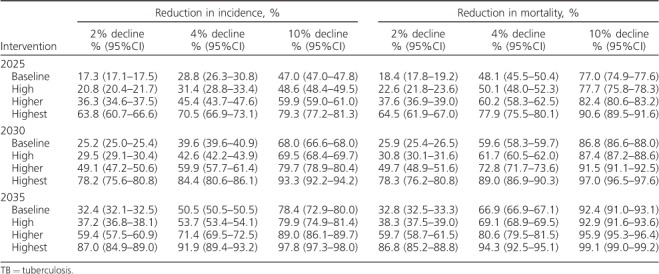
Reductions in TB incidence and mortality achieved by the mass screening and treatment intervention on top of the three background decline scenarios (2%, 4% and 10%)

**Figure A.7:**
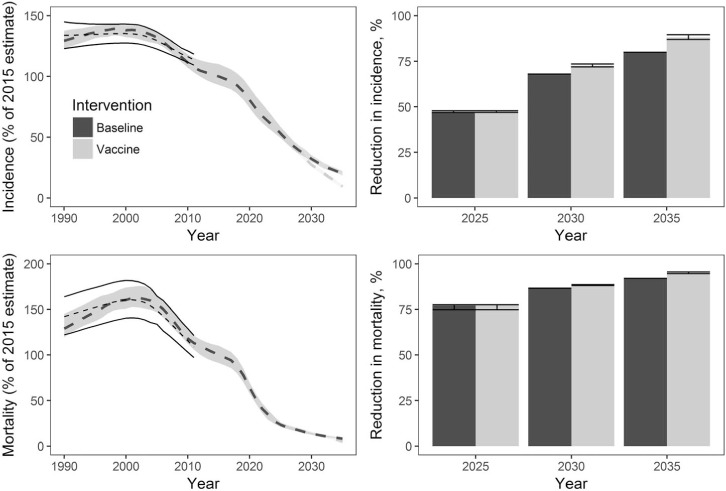
Impact of vaccination in the 10% decline scenario.

**Figure A.8:**
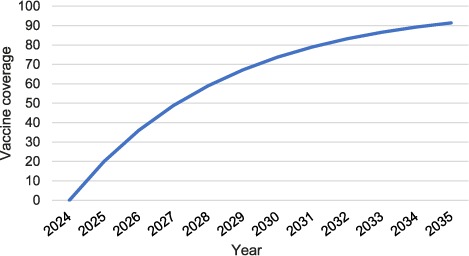
Global vaccine coverage over time assuming 20% of unvaccinated population vaccinated per year.

## Footnotes

^*^The appendix is available in the online version of this article, at http://www.ingentaconnect.com/content/iuatld/ijtld/2018/00000022/00000007/art00006

## APPENDIX

### MODEL STRUCTURE

We developed a deterministic compartmental model of global tuberculosis (TB) transmission which is similar in structure to several previously published models.^1^,^2^ The model describes a single randomly mixing population, stratified by human immunodeficiency virus (HIV) status but not by age. The model structure is shown in Figure A.1. Model parameters (see Table A.1) are based on previously published estimates where possible.

Susceptible (*S*) individuals are infected at a rate of λ =β(*I*+*eN*), where β is the effective contact rate and *I* is the number of smear-positive TB cases, *N* is the number of smear-negative TB cases and *e* is the relative infectiousness of smear-negative TB cases. A proportion α develop primary TB; of these, a proportion σ are smear-positive (*I*), 1-σ are smear-negative (*N*). Of those infected, 1-α become latently infected and can progress to TB at rate ν or can be re-infected. A proportion of those re-infected (α[1*-x*]) develop exogenous TB, where *x* defines the level of protection conferred by a previous infection.

Individuals with smear-positive TB disease are detected at rate *k*, and those with smear-negative disease at a lower rate, given by *dk*. A proportion of these (τ) complete treatment and move to the Treated/Recovered class (*R*). Individuals can also recover naturally at rate *r*_*NAT*_ and also enter the Treated/Recovered class. No distinction is made between individuals who recover naturally or after treatment. Treated/Recovered individuals may relapse to active TB at rate *r*_*REL*_. This rate is assumed to be higher than the rate of reactivation from the latent state, reflecting the increased risk of TB in individuals with a previous history of disease. Treated/Recovered individuals may also be re-infected at rate λ. A proportion α(1-*x*) of these develop exogenous TB (where *x* defines the level of protection conferred by a previous infection and is assumed to be equivalent to the protection in the latent state); the remainder remain in the Treated/Recovered state. Detected cases who do not complete treatment (1-*k*) remain in the diseased state. Prevalent TB cases experience an increased risk of mortality that depends on smear status and HIV status.

The model was implemented using R (R Computing, Vienna, Austria).^8^

#### Model equations

*Drug-susceptible patients*

*Patients with latent tuberculous infection*

*Patients with smear-positive tuberculosis*

*Patients with smear-negative tuberculosis*

*Recovered patients*

where *T* is the total population, super/sub-script *H* refers to HIV-positive parameters and variables, superscript *A* indicates parameters adjusted for antiretroviral therapy (ART) coverage in HIV-positive individuals (see below) and *H*(*t*) is the HIV incidence at time *t*. All other parameters are listed in Table A.1.

#### Human immunodeficiency virus and antiretroviral therapy

The model is stratified by HIV status. HIV is not modelled dynamically, rather HIV-infected individuals become HIV-positive at a time-dependent rate determined by global estimates of HIV incidence.^9^ Individuals are assumed to mix randomly irrespective of HIV status, and the rates of infection with HIV and TB are assumed to be independent of TB and HIV status, respectively. HIV increases the risk of developing primary TB and the rate of reactivation of latent tuberculous infection (LTBI), reduces the protection provided by previous infection, reduces the rate of self-cure and increases the rate of relapse from the recovered/treated state. In addition, an increased proportion of HIV-positive cases develop smear-positive TB. HIV-positive individuals experience higher TB-associated mortality and additional HIV-associated mortality. These effects are captured using HIV-specific parameters where appropriate (Table A.1).

The impact of ART is included by reducing HIV-specific mortality, HIV-positive TB mortality, the probability of developing primary disease and the rate of reactivation as follows:

where ART(*t*) is the proportion of the HIV-positive population on ART at time *t* and ρ is the RR for TB when on ART. Superscript *H* indicates HIV positive parameter values, and superscript *A* indicates ART-adjusted parameter values in HIV-positive individuals.

#### Multidrug resistance

Drug resistance is not modelled explicitly. Instead, a constant number of multidrug-resistant TB (MDR-TB) cases is assumed, equal to 3.7% of all TB cases in 2015.^10^ These are assumed not to benefit from treatment or isoniazid (INH). Therefore, as drug-susceptible TB prevalence falls, MDR-TB makes up a larger proportion of all TB.

#### Demographics

Global population estimates and predictions up to 2035 were obtained from the UN Population Division.^3^ The model was fitted to these estimates by fixing the life expectancy to 67 years^3^ in 2005–2010 and allowing the birth rate to vary.

#### Case detection rate and treatment success

Data on the case detection rate (CDR) and treatment success percentage were obtained from the World Health Organization (WHO) Global tuberculosis report, 2012.^10^ The estimated values from 1990 to 2011 are shown in Figure A.2. As the CDR represents the probability of detection of an incident case, we transformed the CDR to a detection rate, *k*, which can be applied to the pool of prevalence, where *k* = 1 − *e*^*CDR*^.^5^ Due to the difficulty in estimating CDR,^11^ we incorporated uncertainty in the detection rate in our model by allowing the magnitude of *k* to vary between 1 and 3 times the estimated value and by allowing the temporal trend in CDR to be right-shifted by 0–3 years.

#### Model initialisation

The model was initialised in 1700 with a single smear-positive case introduced into a susceptible population with case detection and treatment introduced in 1950 and scaled up to 1990 levels over a 40-year period. This long initialisation ensured that equilibrium was reached pre-1950 and allowed simulation of a realistic epidemic up to 1990.

### CALIBRATING THE MODEL TO GLOBALTB TRENDS (1990–2011)

The model was calibrated to WHO estimates of incidence and mortality by HIV status from 1990 to 2011 using an acceptance-rejection sampling approach to capture uncertainty in the model parameters.^10^ In summary, previous distributions were specified for the model parameters based on previously published estimates (Table A.1). Parameter sets were drawn from uniform distributions using Latin hypercube sampling and the model was run for each parameter set. Runs which lay within the uncertainty bounds of incidence and mortality estimates were accepted. Due to perceived underrepresentation of the uncertainty in the estimates of HIV-positive TB incidence and mortality, we increased the uncertainty bounds on these data sets by a factor of 5. Sufficient parameter sets were drawn to obtain 100 accepted parameter samples. The resulting outputs provide a set of natural history parameters consistent with the estimated trends in TB incidence and mortality. Figure A.3 shows the 100 accepted model runs.

### FITTING BASELINE DECLINES IN TB INCIDENCE AND MORTALITY (2015–2035)

Three projections for trends in TB incidence and mortality in the absence of the intervention were considered. These trends were generated using the statistical model described in the main text, assuming a given annual reduction in incidence and CFR are reached by 2025. In Projection 1, the annual decline in incidence and CFR remains at respectively 2% and 16%; in Projection 2, the annual decline reaches 4% by 2025 and the CFR declines to 10% by 2025; in Projection 3, the annual decline reaches 10% by 2025 and the CFR declines to 6.5% by 2025. From 2025 to 2035, all values remain constant.

The transmission model was fitted to these trends by allowing the rate of case detection to increase by a factor *b*_*i*_^*CDR*^ each year and allowing the effective contact parameter (*β*) and the rate of reactivation (*ν*) to decline by a factor *b*_*i*_^*TB*^ each year, where subscript *i* indicates the year. The increase in case detection approximates a continued improvement in health services, while the contact parameter and reactivation rate are assumed to decline due to general improvements in socio-economic factors. Because the relative impact of improving socio-economic conditions on transmission and reactivation is unclear, we allowed the relative reduction in *β* and *ν* to differ when fitting the model for each accepted parameter set as follows:

- Sample *a* from U[0,1]
- Calculate  and  then

where *a*=1 corresponds to a reduction in the effective contact parameter (*β*) only and *a*=0 corresponds to a reduction in reactivation (*ν*) only.

The best fitting values of *b*_*i*_^*CDR*^ and *b*_*i*_^*TB*^ were estimated by minimising the sum of squared residuals between the model output and the predicted trends from 2016 to 2025 using a downhill-simplex (Nelder-Mead) optimisation algorithm. Figure A.4 shows the results of fitting the transmission model to these declines for each set of accepted parameters.

### INTERVENTIONS

#### Mass screening and treatment (2015–2035)

The hypothetical intervention (Figure A.5) consisted of mass screening for LTBI (using the tuberculin skin test or an interferon-gamma release assay), followed by diagnosis of active disease (using a test such as Xpert^®^ MTB/RIF; Cepheid, Sunnyvale, CA, USA), treatment for active disease and preventive therapy. HIV-negative individuals were screened for LTBI and, if positive, tested for active disease. Those diagnosed as TB cases were offered short-course chemotherapy, while those testing negative were given 6 months of isoniazid. HIV-positive individuals were tested directly for active disease. Detected cases were offered treatment while those testing negative were given 6 months INH.

Detected cases may be lost to follow-up before starting treatment or receive incomplete treatment. Those successfully treated are moved to the treated class. Those receiving preventive therapy are moved to parallel ‘on therapy’ categories. While on therapy, individuals experience some level of protection from TB disease (but not infection). Following completion of therapy, a proportion (γ) is assumed to have been ‘cured’ of their LTBI and move to the drug-susceptible category, and the remainder remain infected. Both protection and ‘cure’ vary by HIV status. Once screened, individuals are not eligible to be screened again for 5 years (on average).

Three intervention scenarios were considered: in the ‘high’ scenario, 5% of the eligible population is screened each year and 10% of those starting INH complete therapy; in the ‘higher’ scenario, 10% of the eligible population is screened each year and 50% of those starting INH complete therapy; in the ‘highest’ scenario, 20% of the eligible population is screened each year and 90% of those starting INH complete therapy. Table A.2 shows the intervention parameters. The intervention was simulated for each set of accepted parameters shown in Figure A.3. Where distributions are given for the intervention parameters in Table A.2, these were sampled independently for each simulation to also capture uncertainty in the intervention assumptions.

#### Potential impact of the introduction of a new TB vaccine (2025–2030)

The potential impact of a new pre- and post-exposure vaccine was also explored using the transmission model. Vaccine was modelled as ‘take’ such that a proportion of the population (given by coverage × efficacy) are completely protected against TB disease. We assumed that the duration of protection was greater than the time frame considered (2025–2035), and that there was no repeat vaccination over this period (irrespective of whether or not protection was conferred by the vaccine). Vaccine was assumed to be distributed randomly across susceptible, latent and recovered individuals and by HIV status. Active cases were not vaccinated.

Assuming a vaccine efficacy of 60% (50–70%), and uptake in 20% of the unvaccinated population per year, the model was used to estimate the reduction in incidence and mortality that could be achieved by 2035 assuming that the annual decline in incidence reaches 10% by 2025 and the CFR falls to 6.5%.

### ADDITIONAL RESULTS

#### Mass screening and treatment

Figure A.6 shows the trends in incidence and mortality from 1990 to 2035 and the percentage reductions in incidence and mortality from 2015 to 2025, 2030 and 2035 predicted using the transmission model for the 4% baseline scenario (see main text for 2% and 10% figures). Dashed lines (bars) show the median trajectories and shaded areas (error bars) show the 95% range based on simulating the intervention for each of the 100 accepted parameter sets. Table A.3 gives the percentage reductions in incidence and mortality achieved by 2025, 2030 and 2035, compared with 2015.

The 75% target for reduction in mortality by 2025 is not achieved in the 2% scenario for any of the intervention assumptions. In the 4% scenario, the highest level of intervention reduces mortality by 75–80% by 2025. In the 10% scenario (in which the 75% target is reached through the background decline alone), the three levels of intervention predict total reductions in mortality of respectively 75–78%, 80–83% and 89–92%.

#### Vaccine

Assuming that a vaccine with 60% (50–70%) efficacy is introduced in 2025, with 20% of the unvaccinated population vaccinated per year from 2025 to 2035, the results of the transmission modelling suggest that TB incidence could be reduced to 10.4/100 000 (8.7–12.1/100 000) by 2035 (assuming the 10% background decline scenario) and a corresponding decline in TB mortality of 95% (94–96%). Figure A.7 shows the trends in incidence and mortality from 1990 to 2035 and the percentage reductions in incidence and mortality from 2015 to 2025, 2030 and 2035 predicted by the transmission model. Dashed lines (bars) show the median trajectories and shaded areas (error bars) show the 95% range based on simulating the intervention for each of the 100 accepted parameter sets. This level of vaccination corresponds to a global coverage of approximately 90% by 2035 (Figure A.8 shows the vaccine coverage over time).

### LIMITATIONS

The modelling described here was intended to illustrate the potential reductions in TB deaths and incidence that could be achieved with ambitious scale-up of existing and future technologies. We did not attempt to develop a dynamic model including all possible variables influencing the TB epidemic and associated projections of how they could influence TB incidence and mortality in the coming decades. This was ruled out because of the difficulties in accurately specifying and quantifying all of the different (and sometimes conflicting) factors that will influence the TB epidemic and associated model parameters. In addition, while major uncertainties in natural history and intervention effectiveness were incorporated in the dynamic model used for projections of what could be achieved with greater use of current interventions as well as projections of what could be achieved with a new intervention to prevent the development of TB disease in people already latently infected with Mycobacterium tuberculosis, these uncertainties may have been underrepresented. Specific limitations of the models are discussed below.

#### Baseline model

The baseline model includes several simplifications and limitations. It assumes a single global population and therefore does not account for regional differences in TB epidemiology. The impact of the intervention may differ by region depending on the characteristics of the local epidemiology, for example the incidence of HIV or the proportion of disease due to recent transmission.

The model assumes a single homogeneously mixing population and does not incorporate potential heterogeneities in contact patterns or TB risk (except by HIV) and we acknowledge that this is a significant simplification of reality. Such heterogeneities may influence the impact of any intervention, for example if a particularly high-risk group had limited access to the intervention. The reasons to consider a single population were two-fold. First, the aim of this modelling was to provide high-level results of the potential impact that could be achieved which could form part of wider discussions around target setting and not to present precise estimates of future trends in different countries. Second, our approach was limited by the time-frame in which the modelling had to be completed and the challenges involved in building and parameterising multiple models for different geographical settings.

In addition, we did not consider age structure or the differences in paediatric and adult TB. Given the noted differences in infectiousness, smear status and diagnosis of TB in children, this is an important simplification. However, given the aim of the model to provide high-level results that could feed into the consultations at which the targets were agreed, we did not consider it necessary to include age structure in the model. We note that efforts to improve diagnosis and treatment of TB in children are likely to be important in achieving the targets.

The proportion of MDR-TB cases is assumed to be fixed at the current level. Future trends in MDR-TB are not known; however, if prevalence were to increase, the potential impact of the intervention would be reduced.

#### Mass screening for tuberculosis intervention

The modelled intervention was intended to illustrate the potential impact that could be achieved from the aggressive use of existing tools. As such it makes a number of simplifying assumptions. The intervention assumes cases are treated immediately or not at all. On the one hand, this may overestimate the impact of the intervention as it does not allow for on-going transmission between detection and starting treatment. On the other, it does not allow for cases to start treatment after some delay.

We do not account for INH resistance in the model and the potential increase in resistance resulting from the widespread use of isoniazid preventive therapy (IPT). If resistance is increased as a result of the use of INH, the impact of the intervention would be reduced. While we account for uncertainty in both natural history parameters and intervention parameters, the model may not fully capture the uncertainty in the impact of the intervention. In particular, there is considerable uncertainty in the long-term protection provided by INH.

The overall impact of the intervention will be strongly influenced by the availability of resources, tools, and infrastructure as well as the uptake and adherence in the population. We explicitly do not consider when or whether billions of tests for infection or active disease can be produced, procured or delivered to populations. Mass screening of low-risk populations can be resisted by the public, who may have to opt in, and uptake and completion rates of IPT, especially in those not recently exposed to infection or in HIV-negative populations is likely to be low. In addition, if repeated screening rounds are carried out, uptake may decline over time. On balance, given these limitations, subjective judgement and experience, we believe the model may be more likely to overestimate than underestimate the impact of MST.

#### Vaccination

Given the lack of data on the likely performance or mechanism of action of new TB vaccines, we had to make some simplifying assumptions about the characteristics of a potential vaccine. We assumed a vaccine efficacy of 60%, similar to that used in previous modelling studies.^16^ However, there is considerable uncertainty around the likely efficacy of new vaccines. Although we incorporated uncertainty in vaccine efficacy in our analysis (50–70%), population-level impacts will depend strongly on the assumed value.

We assumed that the vaccine would work equally effectively in exposed and unexposed individuals. If a vaccine is only effective in one of these groups, the likely impact would be lower. Given estimated levels of M. tuberculosis infection, a pre-exposure vaccine would likely have significantly lower impact over this time horizon. We also assumed that vaccine protection is constant over the modelled time horizon. In reality, vaccine protection is likely to wane over time and we may therefore overestimate the impact. We also assumed that the vaccine efficacy would not vary by age, HIV status or any other individual characteristic.

Finally, the timing and scale up of introduction will have significant impacts on the ability to reach the estimated levels by 2035. If a new vaccination is not available until a later date or the effective coverage is reached more slowly, the impact achieved by 2035 would likely be lower.
